# Association between parity and pregnancy-associated tumor features in high-grade serous ovarian cancer

**DOI:** 10.1007/s10552-024-01876-2

**Published:** 2024-04-05

**Authors:** Camilla Sköld, Sara Corvigno, Hanna Dahlstrand, Gunilla Enblad, Artur Mezheyeuski, Inger Sundström-Poromaa, Karin Stålberg, Anna Tolf, Ingrid Glimelius, Anthoula Koliadi

**Affiliations:** 1 Department of Immunology, Genetics and Pathology, Uppsala University, Uppsala, Sweden; 2 Department of Oncology-Pathology, Karolinska Institutet, Stockholm, Sweden; 3 Vall d’Hebron Institute of Oncology, Barcelona, Spain; 4 Department of Women’s and Children’s Health, Uppsala University, Uppsala, Sweden; 5 Division of Clinical Epidemiology, Department of Medicine, Karolinska Institutet, Stockholm, Sweden

**Keywords:** Ovarian cancer, Parity, Progesterone receptor, Tissue micro array

## Abstract

**Purpose:**

High-grade serous ovarian cancer (HGSC) is the most common ovarian cancer subtype. Parity is an important risk-reducing factor, but the underlying mechanism behind the protective effect is unclear. Our aim was to study if the expression of hormones and proteins involved in pregnancy were affected by the woman’s parity status, and if they may be associated with tumor stage and survival.

**Methods:**

We evaluated expression of progesterone receptor (PR), progesterone receptor membrane component 1 (PGRMC1), relaxin-2, and transforming growth factor beta 1 (TGFβ1) in tumor tissue from 92 women with HGSC parous (*n* = 73) and nulliparous (*n* = 19). Key findings were then evaluated in an independent expansion cohort of 49 patients. Survival rates by hormone/protein expression were illustrated using the Kaplan–Meier method. The independent prognostic value was tested by Cox regression, using models adjusted for established poor-prognostic factors (age at diagnosis, FIGO stage, type of surgery, and macroscopic residual tumor after surgery).

**Results:**

HGSC tumors from parous women were PR positive (≥ 1% PR expression in tumor cells) more often than tumors from nulliparous women (42% vs. 16%; *p*-value 0.04), and having more children was associated with developing PR positive tumors [i.e., ≥ 3 children versus nulliparity, adjusted for age at diagnosis and stage: OR 4.31 (95% CI 1.12–19.69)]. A similar result was seen in the expansion cohort. Parity status had no impact on expression of PGRMC1, relaxin-2 and TGFβ1. No associations were seen with tumor stage or survival.

**Conclusion:**

Tumors from parous women with HGSC expressed PR more often than tumors from nulliparous women, indicating that pregnancies might possibly have a long-lasting impact on ovarian cancer development.

**Supplementary Information:**

The online version contains supplementary material available at 10.1007/s10552-024-01876-2.

## Background

High-grade serous ovarian cancer (HGSC) is the most common ovarian cancer subtype, with a five-year overall survival of less than 50% [1, 2]. History of childbirth lowers the risk of epithelial ovarian cancer, and the risk is further reduced with each additional childbirth [3]. The protective affect against HGSC seems to be more pronounced with a full length-pregnancy [4]. The underlying mechanism behind the protective effect has not been revealed [5]. One of the hypotheses proposed, the cell clearance hypothesis, is that high progesterone levels during pregnancy induces clearance of premalignant cells via apoptosis [6]. This hypothesis is not fully explored, but there are preclinical indications that progesterone, as well as synthetic progestin, has pro-apoptotic or growth-inhibiting effect on ovarian and fallopian tube cells [7–12]. In line with this, high-progestin formulations of oral contraceptives seem to have a stronger risk-reducing effect on ovarian cancer than low-dose oral contraceptive formulations [13].

Based on previous findings, we hypothesized that high levels of progesterone, or possibly other hormones, during pregnancy would impact on hormone receptor/protein expression in the developed tumor of a parous woman, even many years after her childbirths. Progesterone receptor (PR), progesterone receptor membrane component 1 (PGRMC1), relaxin-2, and transforming growth factor beta 1 (TGFβ1) are hormonal receptors/proteins that increase in late pregnancy. The mechanism by which these hormones and proteins affect tumor biology in women that later develop HGSC is not fully understood. In this pilot study, we investigated if parous and nulliparous women developed HGSC tumors with different PR, PGRMC1, relaxin-2 and TGFβ1 expression. Although parity itself has no impact on prognosis in HGSC [14], we studied if different expression of the studied receptors/proteins was related to survival. Key findings were then evaluated in an independent expansion cohort.

## Methods

### Study population

#### Discovery cohort

Our discovery cohort of patients was identified in the Swedish cancer register, and all patients diagnosed with ovarian cancer, fallopian tube cancer and primary peritoneal carcinoma (ICD-O-3: C56.9, C57.0, C48.1, C48.2) in Stockholm county 2002–2006 were screened for eligibility [15]. Patients had to be 18 years or older, diagnosed with ovarian cancer with high-grade serous histology, have disease stage IIC–IV (according to International Federation of Gynecology and Obstetrics, FIGO, 1988), and have available tumor tissue from biopsy or surgery performed before chemotherapy was started. Women were excluded if they had been diagnosed with any previous malignancy (other than carcinoma in situ or basalioma), had received prior chemotherapy, or were diagnosed at autopsy. All cases were confirmed as HGSC by a specialized gynecological pathologist by reviewing the tumor slides. Clinical data were obtained from medical records. Parity status was available for all but one patient. A flowchart on inclusions and exclusions can be found in Supplementary Figure 1.

#### Expansion cohort

To validate results from our discovery cohort, we used a cohort of ovarian cancer patients included in U-CAN, a collection of biomaterials and clinical information from adult cancer patients in Uppsala [16]. All patients were diagnosed with ovarian cancer, fallopian tube cancer and primary peritoneal carcinoma of FIGO stage I-IV in 2010–2016 (according to FIGO, 1988), and all cases were reviewed by a gynecologic pathologist. Patients with other histology than high-grade serous were excluded.

### Tissue microarray (TMA) construction and staining

A TMA was constructed with tumor material (from primary tumor or implantation metastasis in omentum or peritoneum) from the chemo-naïve patients in the discovery cohort [15]. In brief, formalin-fixed paraffin-embedded tumor tissue was stained with hematoxylin and eosin and representative tumor tissue was selected. At least two one mm punches were taken from each patient. Similar technics were used to create the TMA for the expansion cohort. The TMA blocks from the discovery cohort were cut and 4 μm sections were stained using Anti-PR Dako PgR 636 (PR), Anti-PGRMC1 Sigma-Aldrich, HPA002877 (PGRMC1), Anti-Relaxin-2/RLN2, Abcam, Ab 183505 (relaxin-2), and Anti-TGFB1 antibody, Atlas antibodies, HPA008612 (TGFβ1). The staining was performed using an automated protocol with the DAKO Autostainer Link 48 platform. The TMA blocks from the expansion cohort were cut and stained for PR only, using Anti-PR Dako PgR 636 (PR).

### Scoring and cut offs

Scoring was performed by two independent observers (C.S. and A.K.), blinded to the patient data, and difficult cases were confirmed by a gynecological pathologist (A.T.). TMA cores were excluded if evaluable tumor tissue was missing or if only a few cell clusters (< 50 cells) were present. PR was considered positive if ≥ 1% of the tumor cell nuclei stained positive, as recommended in clinical practice for breast cancer assessments [17] and used by other research groups in studies of ovarian cancer [18]. For PGRMC1, relaxin-2 and TGFβ1, there are no established scoring systems. We scored PGRMC1 and TGFβ1 based on the percentage of positive cytoplasmic staining in tumor cells: negative (0): < 1%, weak (1): 1–24%, moderate (2): 25–49%, and strong (3): ≥ 50% of cells; and intensity of the expression: weak (1), moderate (2) and strong (3). We then calculated a combined score by multiplying percentage of positive cells (grade 1–3) with intensity (grade 1–3), obtaining a score of 1–9. Relaxin-2 was expressed in almost all tumor cells and therefore, only intensity of expression was scored as negative/weak versus moderate/strong. If two or more samples were available from the same patient, the cores were scored independently and the highest expression from each patient was used. Representative examples of immunostaining results can be found in Supplementary Figure 2.

### Statistical analyses

Expression levels of PR, PGMRC-1, relaxin-2 and TGFβ1 from the discovery cohort were compared by parity status using Fisher’s exact test. Expression of the investigated hormones and proteins was also stratified by FIGO stage.

We constructed logistic regression models to estimate odds ratios (ORs) with 95% confidence intervals (CIs) for the association between parity (parous versus nulliparous), number of children (1–2 children and > 2 children) and positive expression of the investigated proteins, adjusted for age at diagnosis and FIGO stage. Decision on the factors to adjust for were analyzed using a casual diagram (directed acyclic graphs, DAG), and since both age and FIGO stage are factors known to affect the tumor biology, these were included. We estimated internal correlation between expression of the investigated proteins using the Spearman two-tailed test.

Cancer-specific survival rate by expression of PR, PGMRC1, relaxin-2, TGFβ1was illustrated using the Kaplan–Meier method, and differences in survival proportions were tested with log rank test.

We assessed differences in proportions of PR expression by parity status in our expansion cohort, using Fisher’s exact test, and pooled the results from the discovery cohort with the results from the expansion cohort. In a sensitivity analysis, we excluded patients with FIGO stage < III. We used Cox proportional hazard models to estimate hazard ratios (HRs) and 95% CI for associations between age at diagnosis, FIGO stage, type of surgery, macroscopic residual tumor after surgery, parity and PR expression and all-cause mortality. Results were adjusted for the prognostic factors age at diagnosis, FIGO stage, type of surgery, and macroscopic residual tumor after surgery. Patients were followed from diagnosis until death of any cause, or end of follow up (January 2020).

*p*-values were considered statistically significant if < 0.05. All analyses were performed using RStudio version 1.2.1335 [19].

## Results

### Discovery cohort

The discovery cohort included 92 patients with a median age at diagnosis of 64 years (65 years in parous women; 61 years in nulliparous women); Table 1.

**Table 1:** Clinical characteristics and receptor/protein expression by parity status in patients diagnosed with high-grade serous ovarian cancer, discovery and expansion cohort

	Discovery cohort		Expansion cohort	
	Parous	Nulliparous	Parous	Nulliparous
	*n* (%)	*n* (%)	*n* (%)	*n* (%)
Total	73 (79.3)	19 (20.7)	39 (79.6)	10 (20.4)
Year of birth				
1918–1939	35 (47.9)	6 (31.6)	4 (10.3)	1 (10.0)
1940–1954	33 (45.2)	11 (57.9)	19 (48.7)	2 (20.0)
1954–1973	5 (6.8)	2 (10.5)	16 (41.0)	7 (70.0)
Age at diagnosis (years)				
36–59	22 (30.1)	7 (36.8)	13 (33.3)	7 (70.0)
60–74	31 (42.5)	9 (47.4)	22 (56.4)	2 (20.0)
≥ 75	20 (27.4)	3 (15.8)	4 (10.3)	1 (10.0)
Mean/median	66/65	63/61	62/62	56/52
Number of children				
1–2 children	46 (63.0)	–	30 (76.9)	–
≥ 3 children	27 (37)	–	9 (23.1)	–
Disease stage^a^				
I	0 (0)	0 (0)	5 (12.8)	2 (20.0)
II	1 (1.4)	0 (0)	4 (10.3)	3 (30.0)
III	54 (74.0)	14 (73.7)	21 (53.8)	1 (10.0)
IV	18 (24.7)	5 (26.3)	9 (23.1)	4 (40.0)
Type of surgery				
Primary debulking	56 (76.7)	16 (84.2)	19 (79.2)	9 (100)
Neo-adjuvant chemotherapy + surgery	11 (15.1)	2 (10.5)	4 (16.7)	0 (0)
No surgery	6 (8.2)	1 (5.3)	1 (4.2)	0 (0)
Missing	0 (0)	0 (0)	15 (38.5)	1 (10.0)
Macroscopic residual tumor after surgery				
Absent	21 (31.3)	2 (11.1)	20 (87.0)	7 (77.8)
Present	46 (68.7)	16 (88.9)	3 (13.0)	2 (22.2)
Survival				
Alive	6 (8.2)	1 (5.3)	21 (53.8)	6 (60.0)
Dead	67 (91.8)	18 (94.7)	18 (46.2)	4 (40.0)
*Ovarian cancer death*	*63 (94.0)*	*17 (94.4)*	*12 (66.7)*	*4 (100)*
*Other causes of death*	*4 (6.0)*	*1 (5.6)*	*1 (5.6)*	*0 (0)*
*Missing information on cause of death*	*0 (0)*	*0 (0)*	*5 (27.8)*	*0 (0)*

^a^Data on stage of disease (according to International Federation of Gynecology and Obstetrics, FIGO)

Cancer-specific survival at five years was 25.3% and at ten years 10.7%. Median follow-up time among living patients was 15 years (range: 13–18 years), and among all patients three years (range: 0.0–18 years).

#### Expression of PR, relaxin-2, PGMRC-1, TGFβ1 by parity status

PR expression in the tumor varied with the woman’s parity status, resulting in positive PR expression in 42% of tumors from parous women versus in 16% of tumors from nulliparous women (*p*-value 0.04, Table 2).

**Table 2:** Receptor/protein expression by parity status in patients diagnosed with high-grade serous ovarian cancer (discovery cohort)

	Parous		Nulliparous		*p*-value^a^
	*n*	%	*n*	%	
Total	73	100	19	100	
Progesterone receptor A/B^b^					
Negative, < 1%	41	58	16	84	0.04
Positive, ≥ 1%	30	42	3	16	
Relaxin-2: intensity^c^					
Negative/low	13	22	3	19	1
Medium/high	58	78	16	81	
PGRMC1: score^d^					
0–2	27	39	5	26	0.47
3–9	43	61	14	74	
TGFβ1: score^e^					
0–2	53	76	12	63	0.42
3–9	17	24	7	37	

^a^*p-*value from Fisher’s exact test

^b^Two cases were not possible to analyze because of too few tumor cells in the slide

^c^Two cases were not possible to analyze because of too few tumor cells in the slide

^d^Three cases were not possible to analyze because of too few tumor cells in the slide

^e^Three cases were not possible to analyze because of too few tumor cells in the slide

*PGRMC1* progesterone receptor membrane component 1, *TGFβ1* transforming growth factor beta 1

This translated into an association between increased number of children and PR positive tumors [i.e., ≥ 3 children versus nulliparity, adjusted for age at diagnosis and FIGO stage: OR 4.31 (95% CI 1.12–19.69); Table 3], stronger with additional childbirths, per birth: OR 1.52 (95% CI 1.06–2.26), Table 3.

**Table 3:** Association between parity status and number of children and receptor/protein expression in patients diagnosed with high-grade serous ovarian cancer, adjusted for age at diagnosis and disease stage (discovery cohort)

	PR^a^		PGRMC1 score^b^		Relaxin-2 intensity^c^		TGFβ1 score^b^	
	OR	95% CI	OR	95% CI	OR	95% CI	OR	95% CI
Nulliparous	1.0	Ref	1.0	Ref	1.0	Ref	1.0	Ref
Parous	2.97	0.94–11.48	0.60	0.17–1.79	0.87	0.18–3.22	0.52	0.17–1.62
1–2 children	2.46	0.73–9.93	0.52	0.14–1.65	1.04	0.20–4.39	0.53	0.16–1.79
≥ 3 children	**4.31**	**1.12–19.69**	0.77	0.19–2.91	0.67	0.12–3.12	0.49	0.12–1.90
*Per child*	**1.52**	**1.06–2.26**	0.92	0.65–1.31	0.90	0.58–1.38	0.80	0.54–1.16

Odds ratio (OR) and 95% confidence intervals (CIs) from logistic regression models. Adjusted for age at diagnosis and disease stage (according to International Federation of Gynecology and Obstetrics, FIGO)

^a^ ≥ 1% positive tumor cells

^b^Score ≥ 3

^c^Medium/high expression intensity

*PR* progesterone receptor A/B, *PGRMC1* progesterone receptor membrane component 1, *TGFβ1* transforming growth factor beta 1

Virtually all tumor cells expressed relaxin-2, but the expression intensity did not differ by parity status (*p*-value 1, Table 2). PGRMC1 score did not differ in tumors from parous and nulliparous women (*p*-value 0.47, Table 2), nor did TGFβ1 score (*p*-value 0.42). Neither relaxin intensity, nor PGRMC1 or TGFβ1 score varied by parity status in adjusted analyses (Table 3). PGRMC1 was expressed in 81% and 95% (*p*-value 0.29) of tumors from parous and nulliparous women, respectively, while TGFβ1 was expressed in 50% and 58% of tumors, respectively (*p*-value 0.72), also not varying by parity status in adjusted analyses (Table 3). Intensity of relaxin expression and score of PGRMC1 and TGFβ1 did not differ between parous women and nulliparous women (*p*-values 0.33–0.63, Tables 2, 3). Expression of PR, relaxin-2, PGRMC1 and TGFβ1 did not vary by FIGO stage (Supplementary Table 1).

#### Correlation between PR, PGMRC-1, relaxin-2, TGFβ1

A moderate correlation between expression of relaxin-2 and PGRMC1 score was seen; correlation coefficient was 0.35 (*p*-value: < 0.01). No correlation was seen between any of the other factors (Supplementary Table 2).

#### Expression of PR, PGMRC-1, relaxin-2, TGFβ1 and survival

**Fig. 1:**
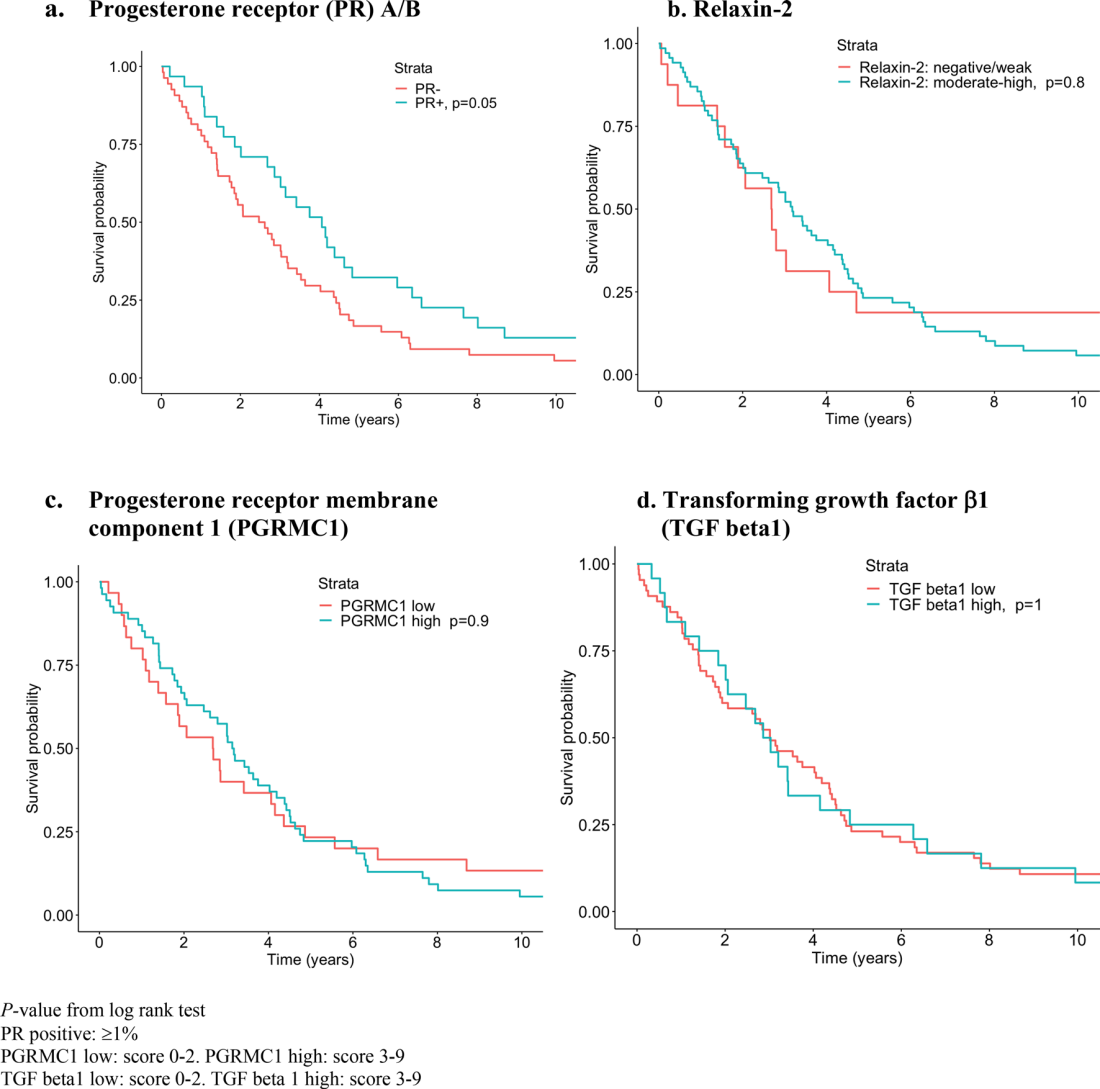
Kaplan Meier curves of cancer-specific survival for patients diagnosed with high-grade serous ovarian cancer by hormone/protein expression, discovery cohort. **a** Cancer-specific survival by progesterone receptor (PR) A/B. **b** Cancer-specific survival by relaxin-2. **c** Cancer-specific survival by progesterone receptor membrane component 1 (PGRMC1). **d** Cancer-specific survival by transforming growth factor β1 (TGF beta1)

A trend to better survival among patients with PR positive tumors was seen (*p* = 0.05). PGRMC1 score, relaxin-2 intensity and TGFβ1 score was not associated with cancer-specific mortality (Fig. 1).

### Expansion cohort

Median age among the 49 included patients in the expansion cohort was 61 years (62 years in parous women; 52 years in nulliparous women); Table 1. Cancer-specific survival at five years was 65.6%; overall survival 57.3%. Median follow-up time among living patients was 5.2 years (range: 3.9–11 years); for all patients 4.8 years (range: 0.2–11 years).

### Expression of PR by parity status

Sixteen of 39 tumors (41%) from parous women had positive PR expression, compared with three of 10 tumors (30%) of tumors from nulliparous women (*p*-value: 0.72, Table 4).

**Table 4:** Progesterone receptor expression by parity status in patients diagnosed with high-grade serous ovarian cancer, expansion cohort and pooled cohorts

	Expansion cohort					Pooled cohorts				
	Nulliparous		Parous		*p*-value^a^	Nulliparous		Parous		*p*-value^a^
	*n*	%	*n*	%		*n*	%	*n*	%	
Total	10	100	39	100		29	100	110	100	
PR A/B, all FIGO^b^ stages										
Negative, < 1%	7	70%	23	59%	0.72	23	79%	64	58%	0.05
Positive, ≥ 1%	3	30%	16	41%		6	21%	46	42%	
PR A/B, FIGO^b^ stage III–IV										
Negative, < 1%	4	80%	18	60%	0.63	20	83%	59	59%	0.03
Positive, ≥ 1%	1	20%	12	40%		4	17%	41	41%	

^a^*p-*value from Fisher’s exact test

^b^International Federation of Gynecology and Obstetrics

*PR A/B* progesterone receptor A/B

#### Pooled cohorts

Patients from the two cohorts were then pooled in an attempt to increase sample size. In this combined cohort, 46 of 110 tumors (42%) from parous women had positive PR expression, compared with six of 29 tumors from nulliparous women (21%), *p*-value: 0.05 (Table 4). When restricting the analysis to only FIGO stage III–IV, 41 of 100 tumors (41%) from parous women expressed PR, compared with four of 24 tumors (17%) from nulliparous women (*p*-value: 0.03).

As expected, older age at diagnosis, more advanced FIGO stage and not having surgery predicted worse overall survival (Table 5), while macroscopic residual tumor after surgery was not associated with prognosis in the adjusted analysis. Parity status had no impact on survival, nor did PR expression.

**Table 5:** Correlation of clinical characteristics and progesterone receptor expression on all-cause mortality in patients diagnosed with high-grade serous ovarian cancer (pooled cohorts)

	Unadjusted		Adjusted^a^	
	HR	95% CI	HR	95% CI
Age at diagnosis (years)				
36–59	1.0	Ref	1.0	Ref
60–74	**1.75**	**1.12–2.74**	**1.72**	**1.05–2.83**
≥ 75	**2.37**	**1.41–4.00**	**2.36**	**1.32–4.21**
Per year	**1.04**	**1.02–1.06**	**1.04**	**1.02–1.06**
Disease stage				
I–II	1.0	Ref	1.0	Ref
III	**4.67**	**1.47–14.85**	**3.76**	**1.11–12.76**
IV	**5.43**	**1.66–17.83**	**5.13**	**1.39–18.96**
Type of surgery				
Primary debulking	1.0	Ref	1.0	Ref
Neo-adjuvant chemotherapy + surgery	1.17	0.66–2.07	1.04	0.56–1.95
No surgery	**3.19**	**1.53–6.66**	**3.05**^b^	**1.40–6.64**
Macroscopic residual tumor after surgery				
Absent	1.0	Ref	1.0	Ref
Present	**2.27**	**1.46–3.53**	1.48	0.90–2.44
Parity				
Nulliparous	1.0	Ref	1.0	Ref
Parous	0.92	0.57–1.47	0.71	0.42–1.22
1–2 children	0.81	0.49–1.32	0.65	0.37–1.15
≥ 3 children	1.23	0.71–2.14	0.85	0.45–1.61
*Per child*	1.08	0.92–1.28	0.98	0.81–1.18
Progesterone receptor A/B pos vs. neg				
Negative, < 1%	1.0	Ref	1.0	Ref
Positive, ≥ 1%	0.69	0.46–1.03	0.79	0.50–1.25

Hazard ratio (HR) and 95% confidence intervals (CIs) from Cox proportional hazards models

^a^Adjusted for age at diagnosis, disease stage (according to International Federation of Gynecology and Obstetrics, FIGO system), type of surgery and macroscopic residual tumor after surgery

^b^Adjusted for age at diagnosis and disease stage (according to International Federation of Gynecology and Obstetrics, FIGO system)

## Discussion

In this study of 92 women diagnosed with HGSC, we found that tumors from parous women expressed PR more often than tumors from nulliparous women. A similar result was seen when an independent expansion cohort of 49 patients was analyzed and patients were pooled in a combined cohort. An American study of 157 women with ovarian cancer of all subtypes [20] found no difference in risk of developing PR positive tumors, defined as > 10% positive staining, between parous and nulliparous women (*p*-value 0.59). This result was confirmed when they updated their analyses by including another 158 cases, now defining PR positive tumors as > 1% positive staining (*p*-value 0.72) [18]. They did however not stratify their analysis by ovarian cancer subtype. The distribution of PR expression differs by ovarian cancer subtype [21], and parity could potentially be differently associated with PR across ovarian cancer subtypes. Hence, a potential association with the HGSC might be hidden by the absent association with the other subtypes.

Our hypothesis was that high levels of progesterone (or possibly other pregnancy hormones) would impact on tumor precursor cells, which could result in different tumor receptor expression in the developed tumor even many years after childbirth. We found that tumors from parous women expressed PR more often than tumors from women who had not given birth. A suggestive explanation could be that high progesterone levels during pregnancy would promote PR expressing cells, leading to a higher likelihood of PR expressing tumors in parous women. Oral contraceptive use gives a protective effect against ovarian cancer up to 30–35 years after discontinuations [22], indicating that exposures of hormones can have a long lasting impact on future tumor development. Our result does not strengthen the cell clearance hypothesis, it does however not contradict it either. If high progesterone levels during pregnancy were to clear premalignant cells, a tumor evolving years later could originate from cells whose malignant transformation began after the pregnancy occurred (and hence did not undergo cell clearance).

Another possible explanation behind higher PR expression in tumors from parous women could be that the epithelium in the distal fallopian tube/ovary differentiates during pregnancy, and that tumor cells thereby are more likely to express PR. This would be in line with results in breast cancer, where pregnancies will cause differentiation of glandular tissue and thereby reduce the breast cancer risk [23]. A possible way to clarify this issue could be by assessing the PR expression in fallopian tube cells from ovariectomies of risk subjects [for examples patients with a Breast Cancer Gene (*BRCA*) mutation] and comparing them with healthy controls.

Unlike the ovarian tumor tissue analysis (OTTA) consortium [21], we did not find an association between PR expression and better survival in patients with HGSC in adjusted analyses. However, the OTTA-consortium only found association between strong PR expression (≥ 50% of tumor cell nuclei) and improved survival, whereas no association was seen with weak PR expression. We found no association between expression of PGRMC1 score, relaxin-2 intensity and TGFβ1 score and cancer-specific mortality neither. The prognostic impact of PGRMC1 is largely unexplored in ovarian cancer, but high serum-levels of relaxin-2 have been associated with tumor progression and adverse survival in several malignancies [24–26], including ovarian cancer [27]. However, previous studies have analyzed the effect of serum relaxin and not immunostaining as we did, which might explain the difference. The lack of association between patient survival and TGFβ1 expression in our study is in line with previous studies [28].

### Strengths and limitations

Our study analyzed a well-defined, Swedish Cancer register-based discovery cohort with detailed clinical data available from computer-recorded patient charts, excluding the risk of recall bias. Moreover, all cases were re-examined by an expert gynecological pathologist, thus minimizing the risk of misclassification and ascertain the HGSC histology. Although the cohort is relatively small, it is homogenous by stage and grade encompassing only specifically reassessed HGSC cases. The rigid criteria of cohort selection limited the number of participants, and restricted our possibilities to further stratify patients. Nonetheless, the aim of creating a highly controlled and homogeneous study cohort limited the number of participants, and restricted our possibilities to find associations and to stratify results in subgroups. Another strength of the study is the validation of our findings in another well-defined cohort, although also limited in number. However, the two cohorts were not quite comparable: the patients were diagnosed during different time periods, and therefore their cancer treatment differed. Most notably, the percentage of patients with no macroscopic disease after surgery was much higher in the expansion cohort. Moreover, mean age among nulliparous women in the expansion cohort was younger.

We did not have information on germline or somatic *BRCA*-mutations and we were thereby not able to examine associations between *BRCA*-mutations and expression of the studied factors. The OTTA-consortium screened a subset of patients for deleterious germline *BRCA1* or *BRCA2* mutations and did not find any association between PR positivity and *BRCA1/2* mutations, suggesting that PR and *BRCA*-mutations are independent factors [21]. We lacked information on hormonal contraceptive use and menopausal hormone treatment. However, prior studies have found that hormonal contraceptive use and/or menopausal hormone treatment have not had an impact on PR expression in tumors [18, 20].

## Conclusions

In our study on women diagnosed with HGSC, we found that tumors from parous women expressed PR more often than tumors from nulliparous women. The result was similar when pooling with an expansion cohort, suggesting that pregnancies could possibly have a long-lasting impact on ovarian cancer development. This indicates the interest of investigating this in confirmatory analyses in a larger, independent cohort. Expression of PR, PGRMC1, relaxin-2 and TGFβ1 had no impact on survival.

### Supplementary Information

Below is the link to the electronic supplementary material.

Supplementary file1 (DOCX 30 KB)

Supplementary file2 (DOCX 7364 KB)

Supplementary file3 (DOCX 16 KB)

## Data Availability

The data analyzed during the current study are not publicly available due to ethical reasons, but are available from the corresponding author on reasonable request.
