# Supplementary material for: Association between parity and pregnancy-associated tumor features in high-grade serous ovarian cancer
Source: Cancer Causes Control. 2024 Apr 5;35(8):1101–9. doi: 10.1007/s10552-024-01876-2 (PMC11266373; doi:10.1007/s10552-024-01876-2)
Supplement: Supplementary file 1 — Supplementary file1 (DOCX 30 KB) [file 10552_2024_1876_MOESM1_ESM.docx]

**Supplementary figure 1. Flowchart on inclusions and exclusions**

1. **Discovery cohort b. Expansion cohort**

199 eligible patients

32 patients: no available tumor tissue

Inclusion criteria

- ≥18 years
- High grade-serous histology
- Biopsy/surgery prior to chemotherapy
- Available tumor tissue
- FIGO stage IIC-IV

31 patients: high grade-serous histologic type not confirmed or missing clinical data

TMA constructed with tumor material from 136 patients

43 patients: <50 tumor cells in TMA cores

After staining: TMA cores from 93 patients available for analyses

Assessed for eligibility: n=401

195 patients: not meeting inclusion criteria

7 patients: no clinical data

6 patients: other subtype than high-grade serous

TMA with tumor material from 55 patients diagnosed with ovarian cancer in Uppsala 2010-2016

After staining: TMA cores from 49 patients available for analyses

FIGO: International Federation of Gynecology and Obstetrics, 1988.

TMA: Tissue Micro Array.
