# Supplementary material for: Association between parity and pregnancy-associated tumor features in high-grade serous ovarian cancer
Source: Cancer Causes Control. 2024 Apr 5;35(8):1101–9. doi: 10.1007/s10552-024-01876-2 (PMC11266373; doi:10.1007/s10552-024-01876-2)
Supplement: Supplementary file 2 — Supplementary file2 (DOCX 7364 KB) [file 10552_2024_1876_MOESM2_ESM.docx]

**Supplementary figure 2. Representative examples of immunostaining results**

Representative examples of immunostaining results for tumors staining weak (1a) and strong (1b) for progesterone receptor; weak (2a) and strong (2b) intensity of progesterone receptor membrane component 1, weak (3a) and strong (3b) intensity of relaxin-2; and weak (4a) and strong (4b) intensity of transforming growth factor β1. Original magnification x200.
