# Supplementary material for: Association between parity and pregnancy-associated tumor features in high-grade serous ovarian cancer
Source: Cancer Causes Control. 2024 Apr 5;35(8):1101–9. doi: 10.1007/s10552-024-01876-2 (PMC11266373; doi:10.1007/s10552-024-01876-2)
Supplement: Supplementary file 3 — Supplementary file3 (DOCX 16 KB) [file 10552_2024_1876_MOESM3_ESM.docx]

**Supplementary table 1. Receptor expression by disease stage in patients diagnosed with high-grade serous ovarian cancer (discovery cohort).**

|  | **FIGO IIC-IIIB** | | **FIGO III** | | **FIGO IV** | | ***p*-value^1^** |
| --- | --- | --- | --- | --- | --- | --- | --- |
|  | **n** | **%** | **n** | **%** | **n** | **%** |  |
| **TOTAL** | 6 | 100 | 63 | 100 | 24 | 100 |  |
| **Progesterone receptor A/B** |  |  |  |  |  |  |  |
| Negative, <1% | 2 | 33 | 43 | 70 | 12 | 50 | 0.07 |
| Positive, ≥1% | 4 | 67 | 18 | 30 | 12 | 50 |  |
| **Relaxin 2: intensity** |  |  |  |  |  |  |  |
| Negative/ low | 1 | 20 | 11 | 18 | 4 | 17 | 0.98 |
| Medium/ high | 4 | 80 | 51 | 82 | 20 | 83 |  |
| **PGRMC1: score** |  |  |  |  |  |  |  |
| 0-2 | 2 | 40 | 21 | 34 | 9 | 38 | 0.94 |
| 3-9 | 3 | 60 | 40 | 66 | 15 | 63 |  |
| **Transforming Growth Factor β1: score** |  |  |  |  |  |  |  |
| 0-2 | 3 | 60 | 43 | 70 | 20 | 83 | 0.38 |
| 3-9 | 2 | 40 | 18 | 30 | 4 | 17 |  |

^1^Stage of disease according to International Federation of Gynecology and Obstetrics, FIGO system of 1988.

^2^*P-*value from Fisher’s exact test.

PGRMC1 = progesterone receptor membrane component 1

**Supplementary table 2. Internal correlation between expression of progesterone receptor A/B (PR), progesterone receptor membrane component 1 (PGRMC1), relaxin 2 and transforming growth factor beta 1 (TGF β1) in patients diagnosed with high-grade serous ovarian cancer* (discovery cohort).**

| **PGRMC1 score** | **Relaxin 2** | **PR** |  |
| --- | --- | --- | --- |
| -0.13 | 0.02 | 0.07 | **TGF β1 score** |
|  | **0.35**** | -0.12 | **PGRMC1 score** |
|  |  | -0.04 | **Relaxin 2** |

*Correlations tested with Spearman two-tailed test.

**=*p-*value <0.01.
